# Targeting S100A12 to Improve Angiogenesis and Accelerate Diabetic Wound Healing

**DOI:** 10.1007/s10753-024-02073-8

**Published:** 2024-07-02

**Authors:** Shitian Qin, Fan Bie, Shuying Chen, Yingbin Xu, Lei Chen, Bin Shu, Fan Yang, Yangzhou Lu, Jialin Li, Jingling Zhao

**Affiliations:** 1 Department of Burns, Wound Repair and Reconstruction, The First Affiliated Hospital of Sun Yat-Sen University, No. 58, Zhongshan 2 Road, Guangzhou, Guangdong Province 510080 PR China; 2 Department of Intensive Care Unit, The First Affiliated Hospital of Sun Yat-Sen University, No. 58, Zhongshan 2 Road, Guangzhou, Guangdong Province 510080 PR China

**Keywords:** S100A12, wound healing, diabetic mellitus wounds, angiogenesis, endothelial function

## Abstract

Long-term inflammation and impaired angiogenesis are thought to be the causes of delayed healing or nonhealing of diabetic wounds. S100A12 is an essential pro-inflammatory factor involved in inflammatory reactions and serves as a biomarker for various inflammatory diseases. However, whether high level of S100A12 exists in and affects the healing of diabetic wounds, as well as the underlying molecular mechanisms, remain unclear. In this study, we found that the serum concentration of S100A12 is significantly elevated in patients with type 2 diabetes. Exposure of stratified epidermal cells to high glucose environment led to increased expression and secretion of S100A12, resulting in impaired endothelial function by binding to the advanced glycation endproducts (RAGE) or Toll-like receptor 4 (TLR4) on endothelial cell. The transcription factor Krüpple-like Factor 5 (KLF5) is highly expressed in the epidermis under high glucose conditions, activating the transcriptional activity of the S100A12 and boost its expression. By establishing diabetic wounds model in alloxan-induced diabetic rabbit, we found that local inhibition of S100A12 significantly accelerated diabetic wound healing by promoting angiogenesis. Our results illustrated the novel endothelial-specific injury function of S100A12 in diabetic wounds and suggest that S100A12 is a potential target for the treatment of diabetic wounds.

**Supplementary Information:**

The online version contains supplementary material available at 10.1007/s10753-024-02073-8.

## INTRODUCTION

Diabetes mellitus (DM) wounds are among the most prevalent complications associated with DM [1]. Poor management of DM wounds may lead to osteomyelitis, amputation, systemic inflammation, even septic shock, and multiple organ failure, causing great physical and mental burdens [2, 3]. DM wounds are a global health challenge associated with massive economic costs for society and public health systems [4].

Cutaneous wound healing involves four successive but overlapping phases: hemostasis, inflammation, proliferation, and remodeling [5, 6]. Persistent inflammation and vascular dysfunction were observed in DM wounds compared to normal healing wounds, leading to delayed wound healing or even nonhealing [7, 8]. Thus, regulation of inflammation and promoting angiogenesis are essential for accelerating DM wound healing [9–11]. Since multiple complex and ambiguous molecular mechanisms are involved in persistent inflammation and vascular dysfunction induced by a high-glucose pathological environment, therapies available in the clinic for promoting DM wound healing have shown little efficacy [12–14].

As one of the S100 families of EF-hand-type calcium-binding cytosolic proteins, S100A12, also known as calgranulin C, was originally thought to be expressed primarily in neutrophils, monocytes, and activated macrophages and is thus linked to innate immune functions and inflammation [15–17]. S100A12 is considered a hallmark of active proinflammatory factors because it is expressed at high levels in many inflammatory diseases, such as lupus nephritis, periodontitis, inflammatory bowel disease and Kawasaki disease [17–20]. In addition, S100A12 expression appears to be high throughout the lesion area of psoriasis according to some studies and contributes to the occurrence and development of psoriasis [21]. Likewise, we demonstrated that the expression and secretion of S100A12 by epidermal cells significantly increased under inflammatory conditions caused by decreased hydration and further activate dermal fibroblasts by binding to S100A12 receptors, including TLR4 (toll-like receptor 4) and RAGE (receptor of advanced glycation end products), thus promoting the formation of hypertrophic scars [22]. Kosaki A et al. found that plasma S100A12 concentration was increased in patients with type 2 diabetes [23]. However, it is still unclear whether S100A12 is involved in microvascular formation and contributes to the initiation and progression of DM wounds.

Krüppel-like factor 5 (KLF5) is a member of the zinc-finger (ZF) transcription factor family, highly expressed in human skin, esophagus, colon, and small intestine [24], regulating a variety of cellular biological processes such as cell proliferation, extracellular matrix deposition, inflammation, and angiogenesis by altering downstream genes and signaling pathway [25–27]. Over the past two decades, KLF5 has been identified as a key factor influencing the progression of vascular diseases [28]. However, there are no studies to report whether KLF5 is involved in the regulation of diabetic wound healing.

In this study, we found that the serum S100A12 concentration was significantly elevated in patients with DM wounds. Exposure of stratified epidermal cells to high glucose led to increased S100A12 expression in epidermal cells, resulting in endothelial dysfunction through the binding of S100A12 to its receptors RAGE and TLR4 on endothelial cells. In addition, the transcription factor KLF5 has been proven to be a direct regulator of the transcription and expression of S100A12 in a high glucose environment. In an *in vivo* study using a diabetic rabbit ear wound model, inhibition of S100A12 significantly improved angiogenesis and accelerated wound healing, demonstrating that S100A12 is a potential target for the treatment of DM wounds.

## MATERIALS AND METHODS

### Stratified Keratinocyte Culture (SKC) and SKC-Endothelial Co-Culture

SKC was performed as our previously published protocol [22]. Briefly, a total of 5 × 10^5^ HaCaT cells were seeded onto the 12-well hanging cell culture inserts (12 mm diameter, 0.4 μm pore size) (Millipore) and cultured in DMEM containing 10% FBS in the first 24 h. Medium in the inner chamber was drained, and the outer chamber medium was replaced into E-medium which consists of 50:50 (v/v) DMEM and DMEM/F-12 supplemented, involving hydrocortisone (0.4 μg/ml), adenine(18 μM), cholera toxin (10 ng/ml), human Apo-transferring (500 ng/ml), bovine pancreatic insulin (500 ng/ml), L-glutamine (4 mM), triiodothyronine (500 ng/ml), epidermal growth factor (EGF, 5 ng/ml), antibiotics (penicillin and streptomycin) and 5% FBS. Changed the E-media every 2–3 days and the stratified epithelium was formed after 10 days. Cells were incubated in a medium containing 30 mM or 5 mM glucose, 30 mM mannitol was served as an osmotic control.

For the SKC-endothelial co-culture model, the inserts with stratified keratinocytes were transferred to 12-well plates where monolayer human microvascular endothelial cell line-1(HMEC-1) were cultured (Fig. 2a) [22].

### Luciferase Reporter Assays

The filtered human S100A12 promoter regions (S100A12-1 ~ 6) were amplified via PCR and subsequently cloned and inserted into the Mul and XhoI restriction sites of the pGL3Basic vector (pGL3-BV). The ligated products were subsequently transformed into competent DH5 alpha cells, which were subsequently plated on lysogeny broth (LB) agar plates supplemented with 100 μg/mL ampicillin (Amp). Promoter-containing pGL3-BV plasmids were transfected into HaCaT cells using Lipofectamine 2000 transfection reagent. Thirty-six hours later, the Dual-Luciferase Reporter Assay System (Promega) was used to measure the luciferase activity of S100A12.

### Chromatin Immunoprecipitation (ChIP) Assays

Chromatin immunoprecipitation (ChIP) assays were used to assess the binding of transcription factors to the promoter regions of S100A12. Formaldehyde was added to the medium for crosslinking of DNA and proteins, and unreacted formaldehyde was neutralized by glycine. The crosslinked chromatin was then sonicated and immunoprecipitated with 10 μg of an anti-KLF5 polyclonal antibody and a normal mouse IgG antibody as a negative control. After elution and purification, the recovered DNA‒protein complexes were analyzed via PCR.

The primer sequences are listed in Supplementary Information Table 1.

### Immunostaining and Western Blot

Normal human skin tissues were obtained from patients with hypertrophic scars who had hypertrophic scar resection and skin grafting. The skin tissues around DM wound were obtained from patients clinically diagnosed with type 2 diabetes mellitus (T2DM) and diabetic ulcer. The patients were informed about the purpose and procedures of the study and voluntarily agreed to provide skin tissue samples. Written informed consent was obtained from all donors, and all protocols were approved by the Ethical Committee of the First Affiliated Hospital of Sun Yat-Sen University. Skin tissues were divided into two groups after washing: one group was stored in liquid nitrogen for western blotting experiment, another group was fixed in formalin for immunofluorescence.

For immunostaining of skin tissues, tissues that fixed in 10% formalin were embedded in paraffin and cut into 5 µm sections. Followed by treatment with antigen retrieval buffer (pH 6.0, DAKO, Carpinteria, CA), the samples were incubated with 1% hydrogen peroxide to quench endogenous peroxidase activity. The samples were incubated with secondary antibody (Vector laboratories, Burlingame, CA, USA) and avidin–biotin complex (Elite ABC kit; Vector Laboratories) after incubation with the primary antibodies. The primary antibodies were as follows: anti-S100A12(abcam,1:100); anti-CD31(abcam,1:200). Cell nuclei were stained by hematoxylin (Sigma), and signals were visualized using 3, 3’-diaminobenzidine (DAB). For immunofluorescence staining of cells, cells were fixed with 4% paraformaldehyde (PFA) and permeabilized with 0.3% Triton X100. Cells were blocked with 10% normal goat serum and incubate with primary antibodies. The primary antibodies were as follows: anti-VE-cadherin(abcam,1:200), anti-S100A12(abcam,1:100) and anti-KLF5(abcam,1:500). Then incubating the sections with Alexa Fluor 594 conjugated secondary antibodies (Thermo Fisher Scientific, Rockford, IL, USA). Cell nuclei were stained with DAPI (4’, 6-diamidino-2 phenylindole, Sigma). Images were taken under a fluorescence microscope (BX51 WI Olympus). NIH Image J software (National Institutes of Health, Bethesda, MD) was used to calculate the signal of total fluorescence intensities after capturing five non-overlapping observation fields, and the average fluorescence intensity for each cell was calculated by dividing the total intensities with the number of cells.

For western blot, proteins from epidermis tissue and HaCaT cells were prepared with RIPA buffer (50 mM Tris, 150 mM NaCl, 1% Triton X-100, 0.1% SDS, and 0.5% sodium deoxycholate, pH 7.5). Equal amounts of protein were separated via SDS‒PAGE and subsequently transferred onto a polyvinylidene difluoride‒nitrocellulose membrane. The membranes were blocked with 5% milk and incubated overnight at 4 °C with primary antibodies as follows: anti-S100A12(abcam,1:1000); anti-KLF5(abcam,1:1000); anti-GAPDH(abcam,1:5000). The membranes were washed and incubated with a horseradish peroxide (HRP)-conjugated secondary antibody (1:5,000 dilution, Vector Laboratories, Burlingame, CA). The protein bands were detected with an enhanced chemiluminescence (ECL) detection kit (GE Healthcare Bio-Sciences, Piscataway, NJ). Using Image J to quantify the results of western blot. Translating the signal of each band into total intensities and the values of signal intensities were employed to show the differences in protein expression.

### Elisa

Secreted S100A12 was detected by enzyme-linked immunosorbent assay (ELISA). Peripheral blood from patients with DM wound and healthy people was collected with a vacuum lancet and then centrifugated for serum preparation. The supernatant of SKC was detected by ELISA. After being blocked with blocking buffer (phosphate-buffered saline (PBS) + 0.05% Tween 20 + 2% bovine serum albumin (BSA)), the cells or serum were incubated with 100 μl of sample mixture in highly bound 96-well plates. A standard curve was generated using the purified human recombinant S100A12 protein. Then, the plates were incubated with 0.25 μg/ml rabbit anti-human S100A12 and HRP-conjugated anti-rabbit antibodies (Jackson ImmunoResearch, West Grove, PA, 1:2000 dilution). The signal was visualized by TMB (3,3′,5,5′-tetramethylbenzidine) substrate (SurModics, Eden Prairie, MN), and the reaction was stopped by 2 M H_2_SO_4_. The optical density was measured with a microplate reader (BioTek Instruments, Winooski, VT) at 450 nm.

### Gene Knockdown

S100A12 knockdown was induced by lentiviral transduction. Short hairpin RNAs (shRNAs) were designed using the RNAi consortium database[29]. The sequences for the S100A12 shRNAs used were showed in Supplementary Information Table 2.

The double-strand shRNA sequences were synthesized by Integrated DNA Technologies (IDT, Coralville, IA). The double-strand shRNA oligos were cloned and inserted into the pLKO.1 puro vector (Addgene, Cambridge, MA), and lentiviruses were prepared according to the protocol provided by Addgene. In the presence of 2 μg/ml puromycin, lentivirus-transduced HaCaT cells were selected. Western blot analysis was used to confirm the effectiveness of the gene knockdown.

### Endothelial Cell Function

HMEC-1 (Human Microvascular Endothelial Cell line-1) was used for endothelial function analysis. Quickly placed cell cryovials in 37 °C water bath and then transferred the mixture to ECM medium for centrifuge. After discarding the supernatant, all the cell suspension was transferred into culture medium at 37 °C, 70%-80% humidity.

#### Scratch Assay

An *in vitro* scratch assay was used to analyze the migration ability of endothelial cells. Drawing horizontal lines evenly every 1 cm on the back of the 6-well plate across the wells. Cells in the logarithmic growth phase was digested into a single-cell suspension with trypsin and then added into plates. After 24 h incubation (37 ℃、5%CO_2_), a gun head perpendicular to the plate was used to scratch along the marking line in the middle of the cells cultured, and the cells were washed twice with PBS to remove the floating cells. Cells were examined by using an inverted microscope (Olympus), and the result was analyzed by Image J software (National Institutes of Health). Cell migration ability was expressed as a percentage of migration distance using the following formula: distance migrated/original wound distance × 100%.

#### Tube Formation Assay

The tube-forming activity of endothelial cells was evaluated via a tube formation assay. Matrigel (BD Biosciences) was plated on precooled 24-well plates and incubated at 37 °C for 30 min. Then, a total of 2 × 10^5^ cells were seeded into the matrix-coated wells and incubated for 8 h at 37 °C. All the cells were fixed and photographed under a light microscope (Olympus). Image J software (National Institutes of Health) was used to quantify the number of tube branches.

#### Cell Viability Assay

MTT assay was used to evaluate the cell viability. Briefly, adding the MTT solution (5 mg/ml) (Sigma) into 96-well plates, and cells were incubated in 37 ℃、5% CO_2_、90% humidity until a purple precipitate was visible. The MTT solution was discarded and the formazan products dissolved in DMSO (Sigma) after reduction to purple formazan. Measuring MTT-formazan crystals at 570 nm with a microplate reader (Multiskan MS, Labsystems, USA). Three replicated MTT assays were done and using the mean values for analysis.

## Animal Experiments

### Protocol for Rabbits with Diabetes

All animal experiments were performed in accordance with procedures approved by the Ethical Committee for the Care and Handling of Experimental Animals of the First Affiliated Hospital of Sun Yat-sen University. New Zealand female rabbits weighting 2–2.5 kg were purchased from the Experimental Animal Research Laboratory at Sun Yat-sen University in China. Housing rabbits individually in cages at suitable temperature (22 °C) and humidity (30–70%) and were provided with ad libitum access to water and standard rabbit food. The rabbits were divided into normal group and DM group (18 rabbits totally). No treatment for the Normal group (6 rabbits) while alloxan was used to induced rabbit diabetes in the DM group (12 rabbits): After mild anesthesia of ketamine, 5% alloxan (dissolved in saline) was intravenously injected into the rabbits via the marginal ear vein at a dose of 100 mg/kg body weight within 2 min. To avoid hypoglycemic shock, 10 ml of glucose (5%) was administered subcutaneously 5 and 10 h after alloxan injection. One week after the first alloxan injection, the rabbits received a second alloxan injection (100 mg/kg) if the fasting blood glucose level (FBG) was < 16 mmol/L to maintain a fasting blood glucose level above > 16 mmol/L[30]. Subcutaneous insulin was necessary if the rabbit FBG was > 20 mmol/L to prevent the occurrence of diabetic ketoacidosis or death. The dose of insulin was as follows: (1) FBG 20 ~ 22.2 mmol/L received 1 U/kg; (2) FBG 22.3 ~ 27.7 mmol/L received 2 U/kg; (3) FBG 27.8 ~ 33.3 mmol/L received 3 U/kg; and (4) FBG > 33.3 mmol/L received 4 U/kg. Blood glucose levels were measured in the morning daily for the first 4 weeks and then weekly.

### Induction of Cutaneous Wounds

To evaluate the rate of wound healing *in vivo*, full-thickness skin wounds were generated on each rabbit ear as described previously[22]. Briefly, seven 6-mm full thickness wounds were made down to the bare cartilage on the ventral side of each rabbit ear using a biopsy punch. The ventral side of the rabbit ear was covered by the semiocclusive dressing Tegaderms (3 M Health Care, St. Paul, MN) to prevent desiccation of the wounds. No treatment was done for normal rabbit ear wounds, half of the rabbits in the DM group were not treated while the other half rabbits were treated as follows: For the S100A12-siRNA treatment (DM + siA12 group), 30 ng/ml S100A12-siRNA-containing plasmids diluted in PBS (Li Rui Biological Technology Co, Ltd, Shanghai, China) was applied to the wounds in one side of ears, and PBS containing vector plasmids was applied to the wounds in the other side of ears (DM + Vec). The efficiency of S100A12-knockdown in rabbit ear wounds was confirmed by RT-PCR analysis. All wounds were bandaged with sterile gauze and treated with the experimental solutions daily. The wound dressings were changed, and digital photographs were taken daily to record wound healing.

### Analysis of Wound Healing

The wounds were digitally photographed at specified intervals. In double-blind conditions, Image-Pro Plus v. 6.0 (Media Cybernetics) was used to analyze wound closure. The wound areas were standardized by comparison with the original wound size, and the healing rate was expressed as a percentage of wound closure using the following formula:

$$\left[\left(\mathrm{Day}\;0\;\mathrm{area}\;-\;\mathrm{day}\;\mathrm n\;\mathrm{area}\right)/\left(\mathrm{Day}\;0\;\mathrm{area}\right)\right]\;\times\;100\%\;\left(\mathrm n=\;0,\;7\;\mathrm{or}\;14\right)$$

The capacity of endothelial cells to form blood vessels was expressed in microvascular density (MVD) calculated by the CD31 immunofluorescence signal. Vessels formed by CD31-positive endothelial cells were counted in a 10 × 20 power field.

### Statistics

Statistical analyses were performed with SPSS software (version 17.0, SPSS, Chicago, IL, USA). All data were represented as mean ± SD. Unpaired Student’s t test was used for two-group comparisons, all data were first subjected to the Shapiro–Wilk test for normality. Two-way ANOVA and One-way ANOVA followed by the Tukey–Kramer’s post hoc test was used for multiple group comparisons. *P* < 0.05 was considered significant in all the experiments.

## RESULTS

### The Expression of S100A12 Increased in the Epidermis and Serum in a High-Glucose Environment

**Fig. 1:**
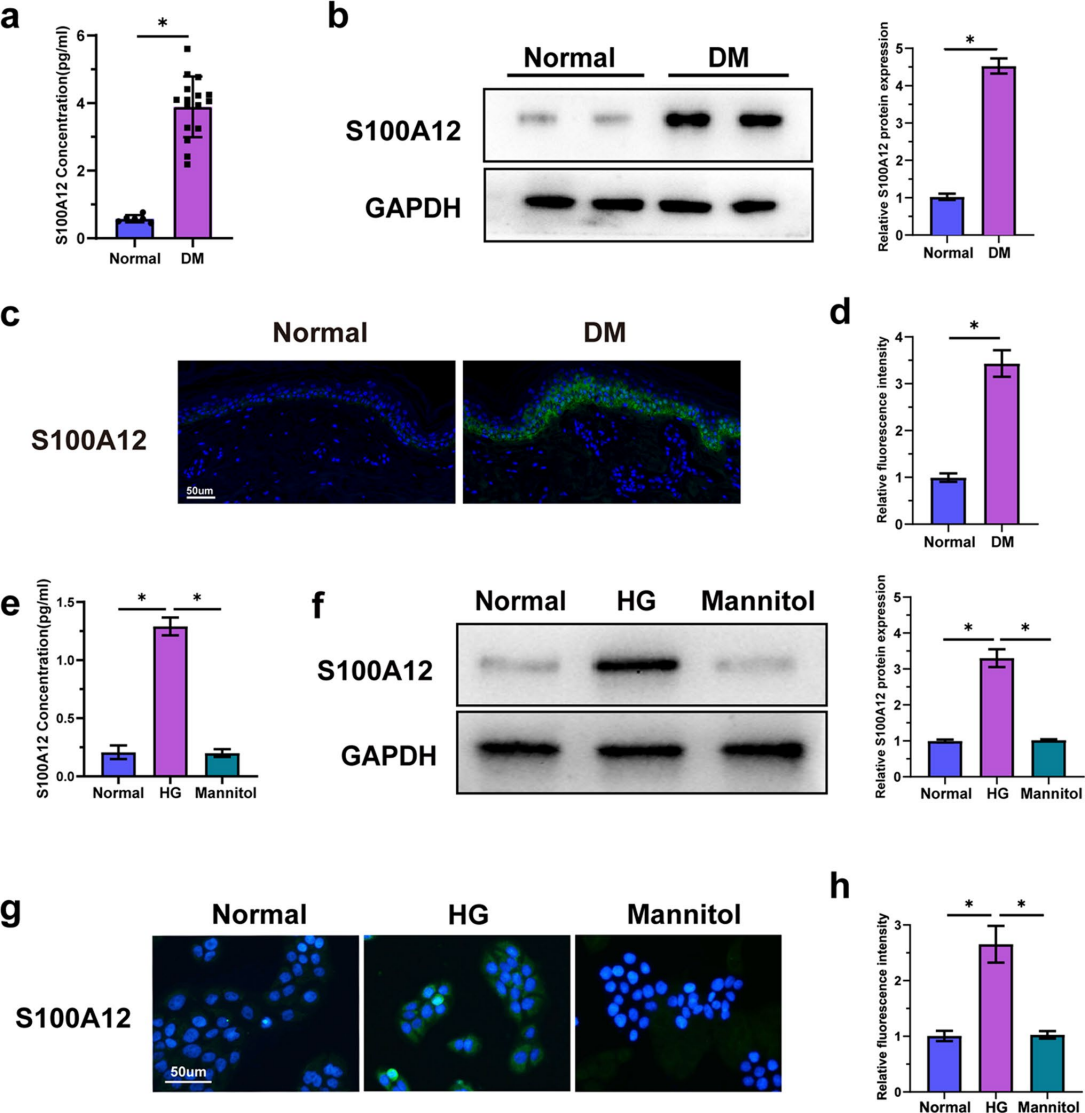
The expression of S100A12 in the epidermis and serum increased in a high-glucose environment. **a** S100A12 concentrations in the serum of healthy people and DM wound patients were determined via ELISA (Normal group *n* = 6, DM group *n* = 16). **b** Western blot analysis of the expression of S100A12 in the normal skin and skin surrounding the DM wounds. The quantification was expressed as fold changes to the normal group (*n* = 3). **c** Immunostaining of S100A12 in the normal skin and skin surrounding DM wounds (nucleus-blue; S100A12-green). Bar = 50 μm. **d** Quantification of the expression of S100A12 in different skin tissues by Image J in (c) (*n* = 7). **e** The concentration of S100A12 in HaCaT cell culture medium under different conditions was detected via ELISA (*n* = 6). **f** Western blot analysis of the expression of S100A12 in HaCaT cell cultured under different conditions. The quantification was expressed as fold changes to the normal group (*n* = 3). **g** Immunostaining of S100A12 in HaCaT cell cultured under different conditions. (nucleus-blue; S100A12-green)** h** Quantification of the expression of S100A12 in (c) (*n* = 7). The data are expressed as the mean ± SD. *P < 0.05, unpaired t test or one-way ANOVA.

The S100A12 concentration was analyzed by ELISA to evaluate its expression in serum. Compared to those in healthy donors, serum S100A12 levels were significantly elevated in DM patients (Fig. 1a). Western blot analysis revealed markedly greater S100A12 expression in the skin around the wounds of DM patients than in that of healthy individuals (Fig. 1b), which was proven by immunofluorescence (Fig. 1c and 1d). In immunofluorescence assay, the epidermal layer of the skin around DM wound showed strong green fluorescence compared to normal skin, demonstrating that S100A12 was predominantly expressed in the epidermis, and expressed at a high level in DM patients (Fig. 1c). To study the relationship between glucose concentration and S100A12 expression in the epidermis, we stratified HaCaT cells, a spontaneously immortalized human keratinocyte cell line (SKC), into three groups. In our model, after 2 weeks of culture to imitate the human epidermis, the cells were stratified and differentiated into multilayer structures in a transwell plate. Culturing the dishes in normal or high glucose (30 mM) environments, and mannitol with the same concentration was used to eliminate the potential impact of osmotic pressure on S100A12 expression and secretion. Compared to the normal environment, significantly higher level of S100A12 expression was detected in the HG medium supernatant by ELISA. However, the results showed no obvious differences between normal and mannitol groups (Fig. 1e). Immunofluorescence intensity of S100A12 in epidermal cells was greater in the high glucose group than in the control group (Fig. 1g and 1h), suggesting that the expression of S100A12 was greater in the HG group. Western blot has further confirmed the immunofluorescence results. (Fig. 1f).

### HG-Induced S100A12 Secretion From the Epidermis Impairs the Functions of Endothelial Cells

**Fig. 2:**
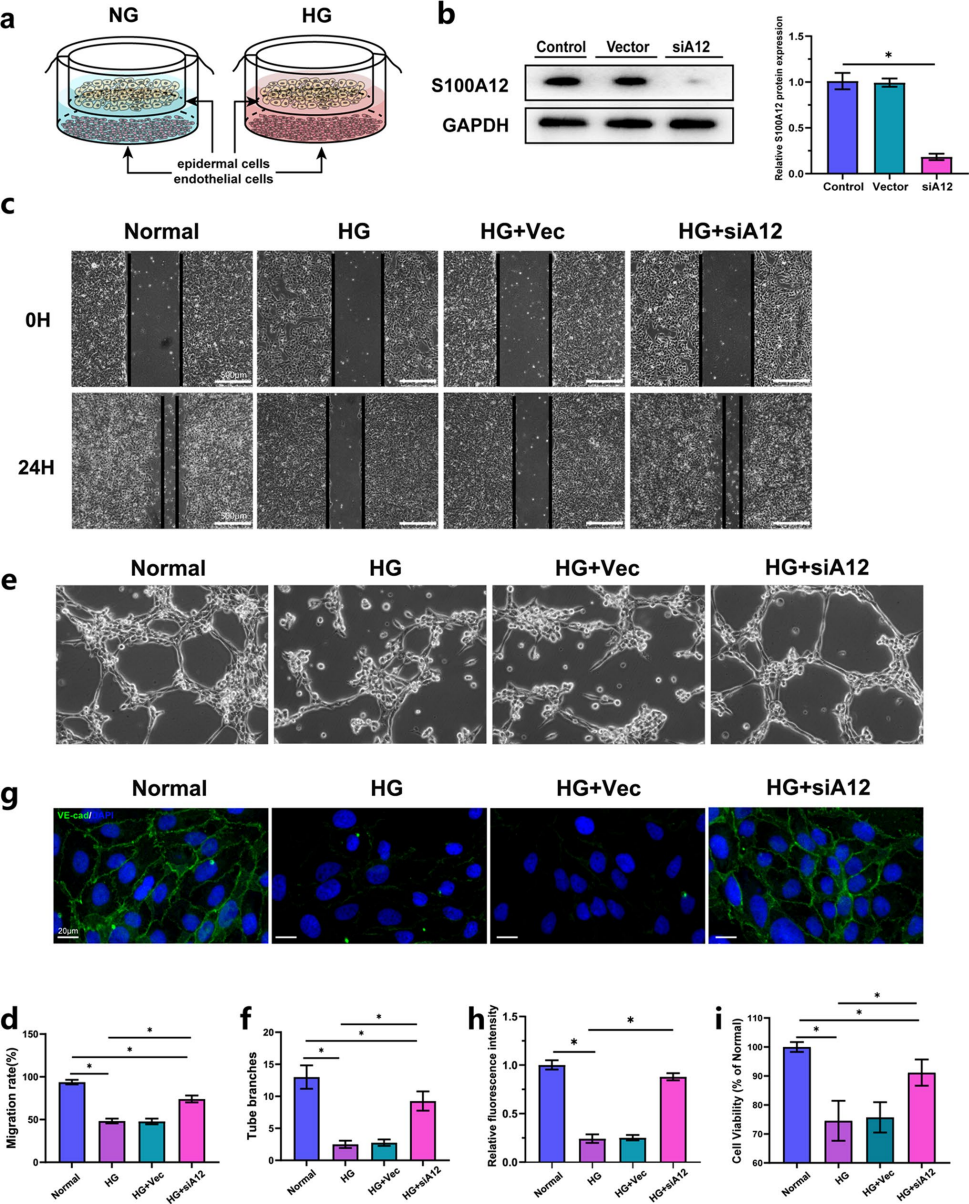
HG-induced S100A12 secretion from the epidermis impairs the functions of endothelial cells. **a** Schematic drawing of SKC-endothelial co-culture model. **b** Western blot analysis was used to verify the efficiency of S100A12 knockdown in HaCaT cell via siRNA. The quantification was expressed as fold changes to the control group (*n* = 3). **c** Migration ability of endothelial cells (a) was determined by a scratch wound healing assay. The vertical bars represent the cell positions after migration. Bar = 500 μm. **d** Quantification of the cell migration rate (*n* = 4). **e** A tube formation assay was used to assess the ability of endothelial cells to form three-dimensional capillary-like structures *in vitro*. **f** Quantification of the tube branches in (e) (*n* = 6). **g** Immunofluorescence staining of VE-cadherin in endothelial cells cultured under different conditions (nucleus-blue; VE-cadherin-green). Bar = 20 μm. **h** Quantification of the expression of VE-cadherin in (g) (*n* = 7). **i** Endothelial cell viability in different conditions was measured by MTT assay (*n* = 6). The data are expressed as the mean ± SD. *P < 0.05, one-way ANOVA. Vec: Vector; siA12: siS100A12.

To investigate the effect of S100A12 secreted from HaCaT cells, we cocultured stratified wild-type and S100A12-knockdown HaCaT cells with endothelial cells (Fig. 2a). Densitometric quantification of the western blot data showed that S100A12 expression was significantly lower following lentivirus-mediated RNA interference in HaCaT cells (Fig. 2b). Motility of endothelial cells and intact intercellular junctions are essential for maintaining vascular homeostasis, which plays a crucial role in wound healing. Migration ability, tube formation ability, barrier function and cell viability were measured to evaluate endothelial functions in our research. Compared to those under normal glucose conditions after 24 h of culture, endothelial migration significantly decreased under high glucose conditions, and the decrease dramatically improved following S100A12 knockdown (Fig. 2c and 2d). Similarly, endothelial cells formed fewer capillary-like tubes in the HG treatment group than in the control group. However, under S100A12-knockdown conditions, the tube formation ability of endothelial cells dramatically improved (Fig. 2e and 2f). Endothelial injury and loss of barrier function are associated with loss of VE-cadherin. Immunofluorescence was used to analyze the distribution of VE-cadherin at junctions between endothelial cells. Along the endothelial intercellular junctions, the protein VE-cadherin under normal glucose conditions exhibited continuous increases in fluorescence intensity. The expression of cell surface-associated VE-cadherin in the HG group decreased significantly, whereas it was upregulated in the S100A12-knockdown treated endothelial cells (Fig. 2g and 2h). Meanwhile, cell viability was reduced in HG condition as assessed by the MTT assay, this alteration was reversed after knockdown of S100A12 (Fig. 2i). These results suggest that S100A12 secreted from epidemic cells may impair endothelial functions directly.

### RAGE and TLR4 Act as Receptors to Mediate S100A12-Induced Endothelial Cell Damage

**Fig. 3:**
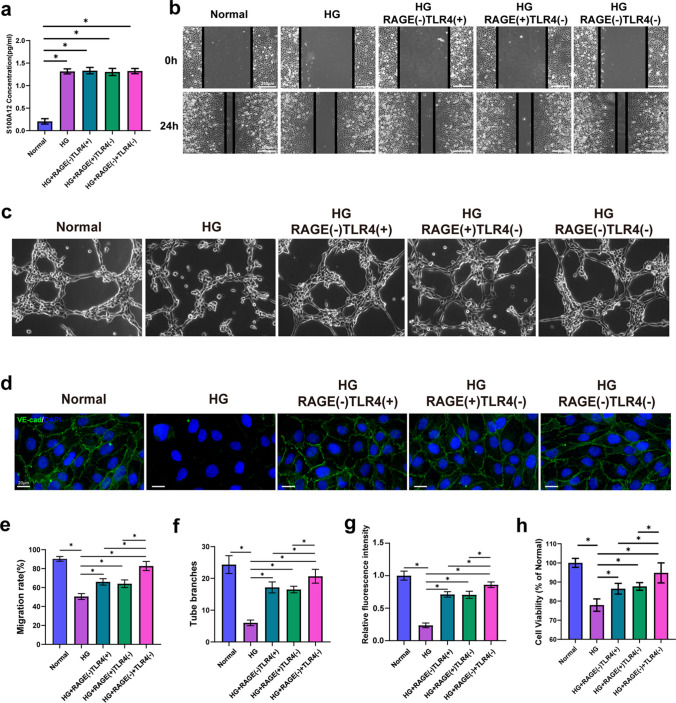
RAGE and TLR4 act as receptors to mediate S100A12-induced endothelial cell damage **a** The concentration of S100A12 in HaCaT culture medium under different conditions was detected via ELISA (*n* = 6). **b** Scratch wound‐healing migration assay. The vertical bars represent the cell positions after migration. Bar = 250 μm. **c** Tube formation assay of endothelial cells. **d** Immunofluorescence staining of VE-cadherin in endothelial cells (nucleus-blue; VE-cadherin-green). Bar = 20 μm (*n* = 6). **e** Quantification of cell migration data in (b) (*n* = 4). **f** Quantification of the tube branches in (c). **g** Quantification of the expression of VE-cadherin in (d) (*n* = 7). **h** Endothelial cell viability in different conditions were measured by MTT assay (*n* = 6). The data are presented as the mean ± SD. *P < 0.05, one-way ANOVA.

Previous studies have suggested that RAGE and TLR4 are the main receptors for the S100A12 protein in the inflammatory process[31, 32]. To investigate whether S100A12 affects endothelial function by acting on these cells, the specific antagonists FPS-ZM1 and TAK-242 were used to inhibit TLR4 and RAGE respectively. ELISA assay results showed significantly increased expression of S100A12 in HG environment, and blocking of RAGE/TLR4 receptors had no impact on S100A12 expression (Fig. 3a). The functions of endothelial cells were impaired under HG conditions, and inhibition of the RAGE or TLR4 receptor with specific antagonists partly abolished this suppression (Fig. 3b-3h). Compared with those in the HG group, the migration ability, tube formation ability, barrier function and viability of endothelial cells in the TLR4- or RAGE-inhibited group improved significantly. Simultaneous inhibition of both receptors resulted in a more significant improvement in endothelial function than inhibition of either receptor alone (Fig. 3b-3h). These data showed that S100A12 may impair endothelial cells function via the TLR4 and RAGE receptors.

### KLF5 Binds S100A12 and Further Enhances its Activity Under HG Conditions

**Fig. 4:**
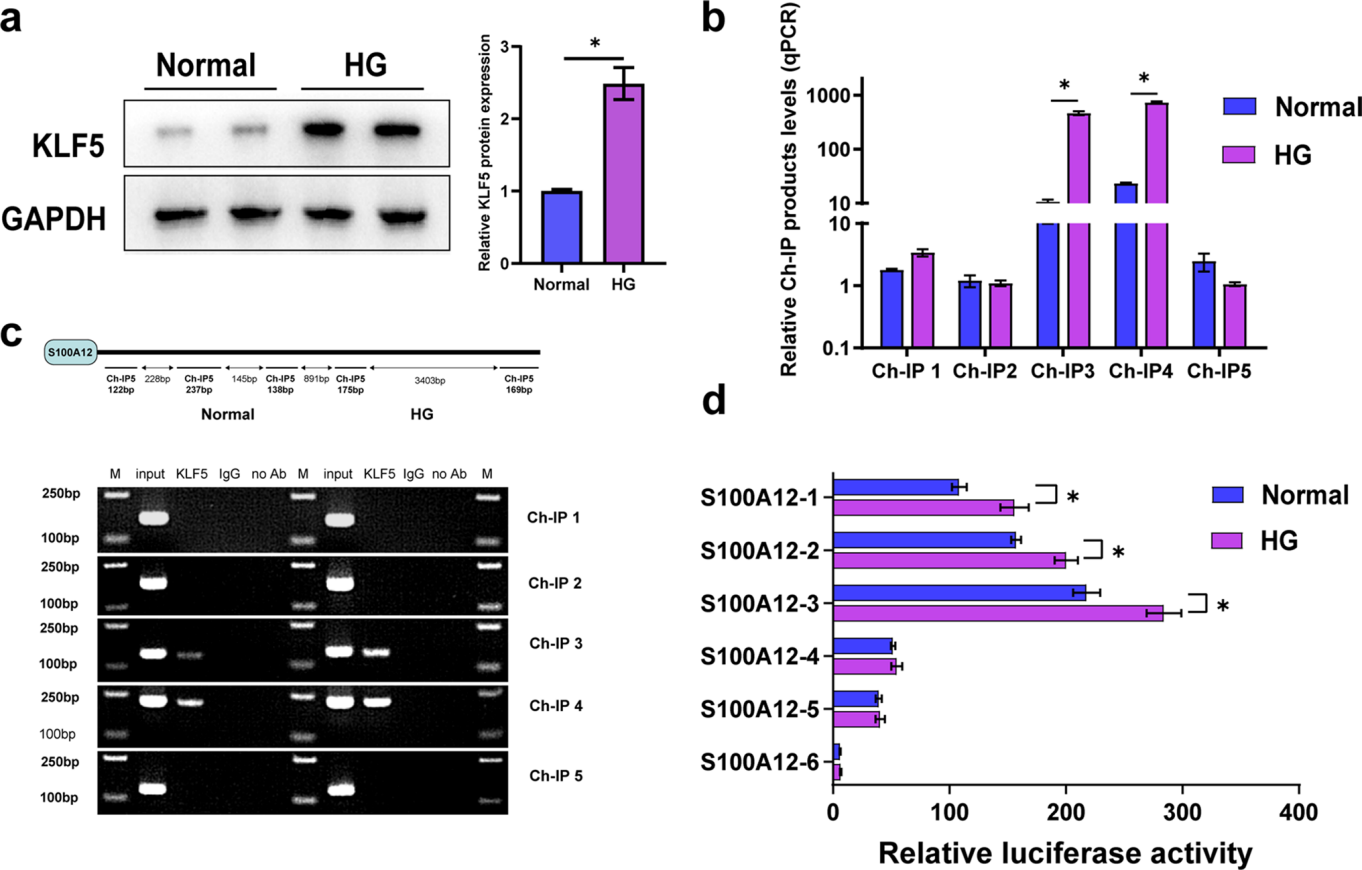
KLF5 binds S100A12 and further enhances its activity under HG conditions. **a** Western blot analysis of KLF5 in HaCaT cells cultured under normal and high-glucose conditions. The quantification was expressed as fold changes to the normal group (*n* = 3). **b** Results of qPCR amplification with the S100A12 primer pair (*n* = 3). **c** Agarose gel electrophoresis analysis of the PCR results with the S100A12 primer pair. **d** A dual luciferase assay was used to detect S100A12 promoter activity in HaCaT cells (*n* = 3). The data are presented as the mean ± SD. *P < 0.05, one-way ANOVA or two-way ANOVA.

To further investigate the upstream regulatory mechanisms of S100A12 expression, we searched Jaspar database for transcription factors that may regulate transcription of S100A12. KLF5 regulates a variety of cellular processes, overexpression of KLF5 in diabetic mellitus mice significantly inhibits endothelial nitric oxide synthase 3 (eNOS3) transcription, thereby hindering endothelial cells proliferation and blunting angiogenesis[26]. In diabetic cardiomyopathy, increased expression of KLF5 was identified as an important factor causing oxidative stress and ceramide accumulation in cardiomyocytes[27]. By consulting in Jaspar database, KLF5 was found to have possible binding sites with S100A12 promoters. Chromatin immunoprecipitation (ChIP) assays were conducted to verify whether KLF5 transcriptionally activates S100A12 in HaCaT cell lines. By consulting the database, we determined the promoter sequence region of S100A12 (-2000 bp ~  + 500 bp) and predicted 11 KLF5 binding sites. For the promoter, five sets of amplification primers (S100A12 ChIPF1/PR1 ~ S100A12 ChIPF5/PR5) were designed. According to agarose gel electrophoresis, specific S100A12 ChIPF3/PR3 and ChIPF4/PR4 bands were amplified with KLF5 antibody-bound DNA as a template (Fig. 4a). Our analysis also showed that KLF5 binds to S100A12 promoters at site 3 (-489 bp ~ -351 bp) and site 4 (-207 bp ~  + 30 bp). In addition, there were significantly greater levels of qPCR products in the HG groups than in the NG groups at both site 3 and site 4 (Fig. 4b).

In addition, to detect S100A12 promoter activity under high-glucose conditions, we transfected HaCaT cells with plasmids containing the S100A12-1 ~ 6 promoter in the HG and NG groups. After 48 h, the protein lysates were prepared and used to measure dual luciferase activity. As demonstrated by previous reports, the luciferase activity of all groups with S100A12 promoters increased by various degrees compared to that of the control group, and these differences were significant (*P* < 0.05). Luciferase activity was significantly greater in the S100A12-1 ~ 3 group under high glucose conditions than under normal glucose conditions (*P* < 0.05), and the S100A12-3 promoter exhibited the highest level of luciferase activity (Fig. 4c). Taken together, these results indicate that under HG conditions, the transcription factor KLF5 has a greater level of binding to the S100A12 promoter, and the activity of the S100A12 promoter increases.

### S100A12 Transcription and Expression are Regulated by KLF5

**Fig. 5:**
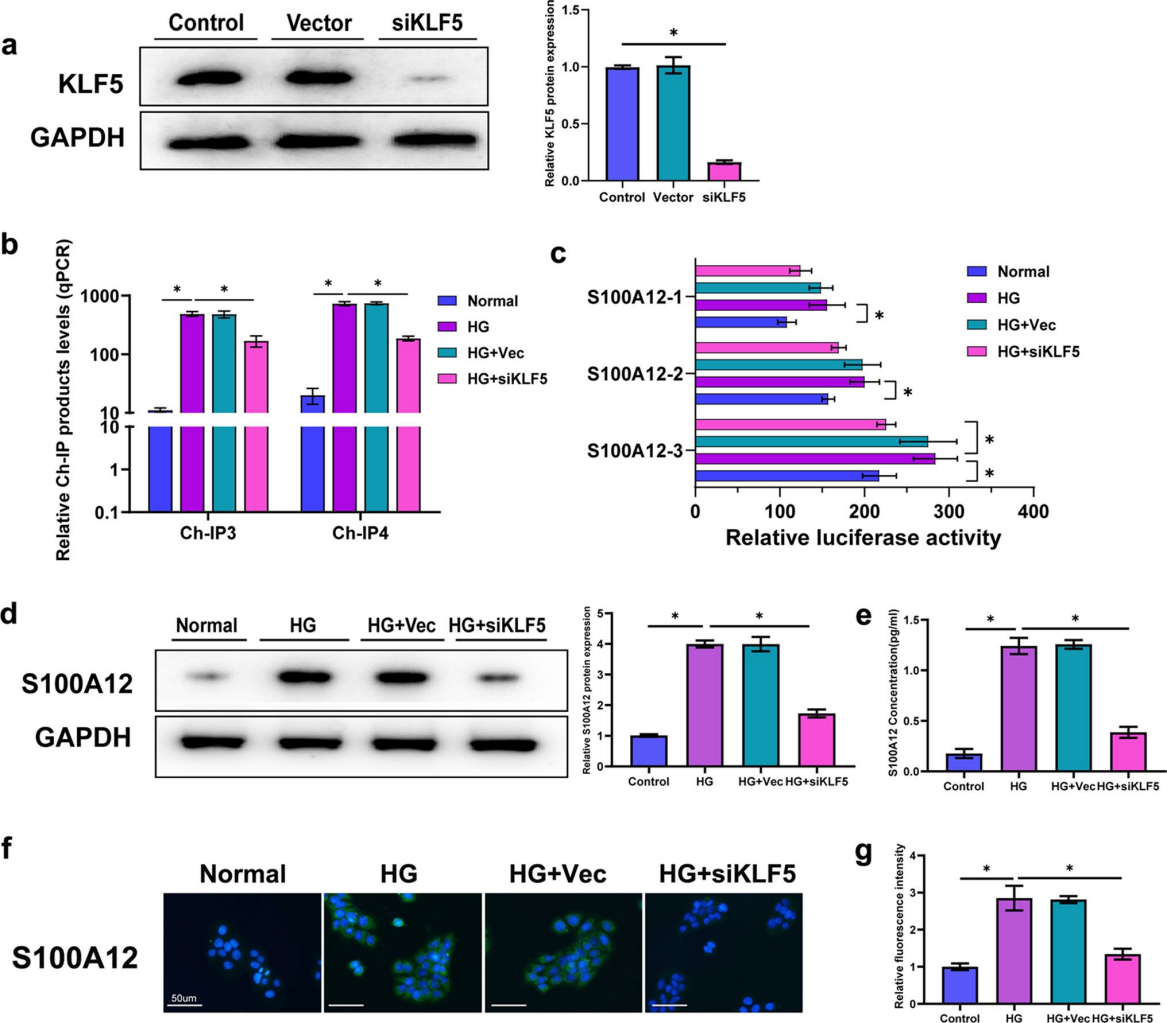
KLF5 inhibition downregulated S100A12 expression. **a** Western blot analysis of KLF5 in HaCaT cells. The quantification was expressed as fold changes to the control group (*n* = 3). **b** Results of qPCR amplification with S100A12 primers (IgG homogenization) (*n* = 3). **c** A dual luciferase assay was used to detect promoter activity (*n* = 3). **d** Western blot analysis of the S100A12 protein. The quantification was expressed as fold changes to the normal group (*n* = 3). **e** The concentration of S100A12 was analyzed by ELISA (*n* = 6). **f** Immunofluorescence staining of S100A12 in endothelial cells (nucleus-blue; S100A12-green). Bar = 50 μm (*n* = 6). **g** Quantification of the expression of S100A12 in (f) (*n* = 6). The data are presented as the mean ± SD. *P < 0.05, one-way ANOVA or two-way ANOVA.

We inhibited KLF5 expression by using a specific siRNA to determine the effect on S100A12 expression. Western blot analysis showed that KLF5 expression was decreased in HaCaT cells after siRNA-KLF5 was introduced (Fig. 5a). The ChIP results suggested that KLF5 knockdown decreased the level of KLF5 binding to the S100A12 promoter in all groups especially group 3 and 4(Fig. 5b). Moreover, the S100A12 promoter exhibited a significantly decreased degree of activity after KLF5 was knocked down (Fig. 5c). Western blot was used to further detect changes in S100A12 expression. The results illustrated that the expression of S100A12 was significantly decreased in the HG condition with KLF5 knockdown (Fig. 5d), which was confirmed by an immunofluorescence assay (Fig. 5f and 5g). The ELISA results were consistent with the above-described results (Fig. 5e).

### Inhibition of S100A12 Promoted Wound Healing in Rabbits

**Fig. 6:**
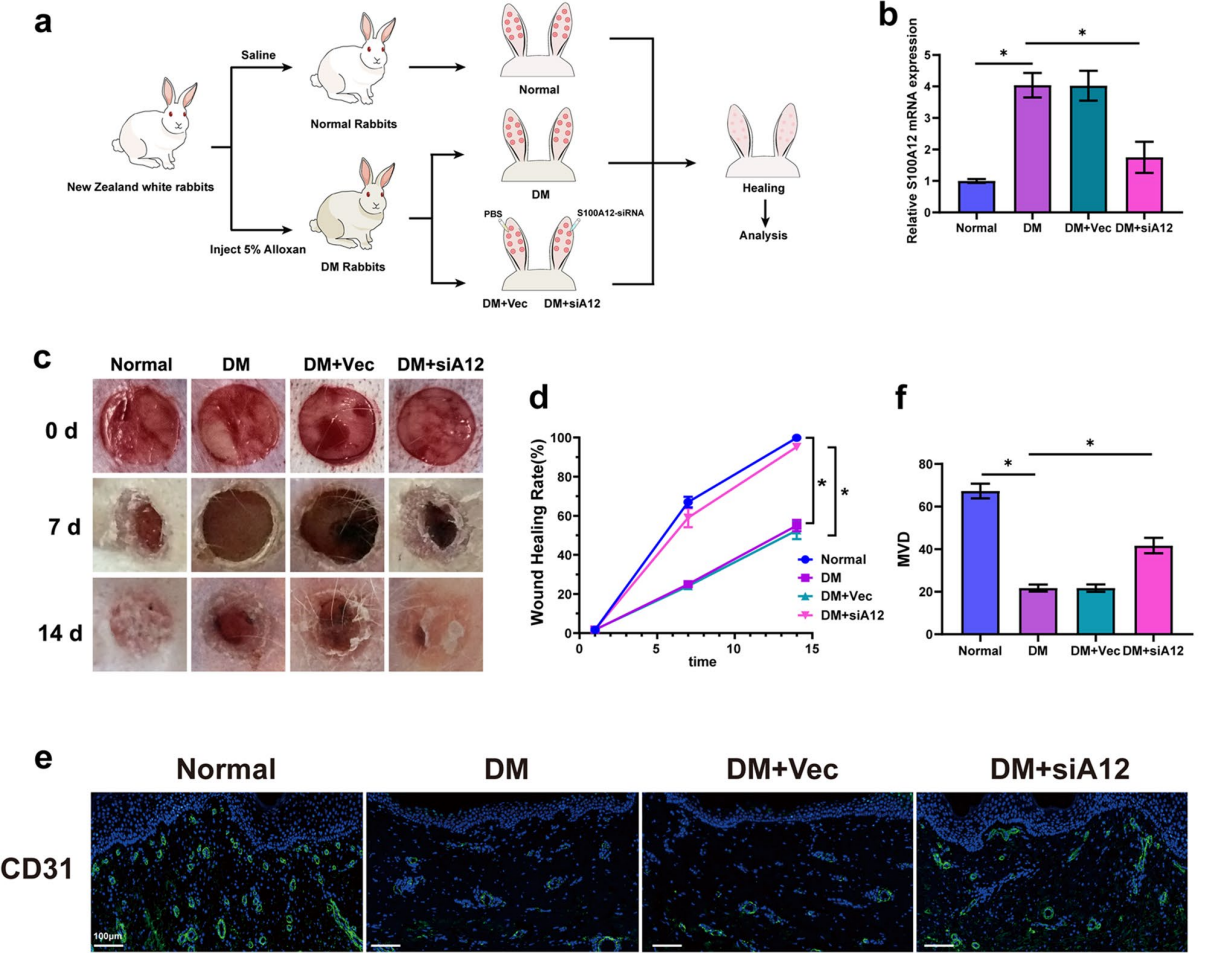
Inhibition of S100A12 promoted wound healing in rabbits. **a** Flow chart of the *in vivo* experimental scheme. (6 rabbits/group, 7 wounds/ear). **b** The efficiency of S100A12-knockdown in rabbit ear wounds was confirmed by RT-PCR analysis (*n* = 6). **c** Representative images of wounds under different conditions at Days 0, 7 and 14. **d** Percentage of the initial wound area during the healing process for different treatments (*n* = 6). **e** Immunofluorescence staining of CD31 in skin sections from different experimental groups (*n* = 6). Endothelial cells were stained in green, using an anti-CD31 antibody. Bar = 100 μm. **f** Microvessel density of wounds in different groups (*n* = 6)**.** The data are presented as the mean ± SD. *P < 0.05.

The effect of S100A12 on endothelial cells and its regulatory mechanism was demonstrated *in vitro* experiments, it is also necessary to demonstrate the feasibility of targeted inhibition of S100A12 in treatment of DM wounds by using animal model (Fig. 6a). The results of RT-PCR analysis confirmed that the expression of S100A12 in rabbit ear wounds decreased after the application of S100A12-siRNA (Fig. 6b). Wound healing tests showed that the healing of wounds in DM rabbits was significantly delayed but improved after S100A12 knockdown (Fig. 6c). On day 7, the wound closure rate was 67% in the control group, while it was only 25% in the DM group and 59% in the S100A12 knockdown group. The wounds in the control group (99%) and the DM + siA12 group (95%) had completely closed by day 14, while almost half of the wound areas in the DM group (45%) remained unhealed (Fig. 6c and 6d).

The microvessel (stained with CD31) count showed that the DM group had markedly lower microvessel density than the control group. After S100A12 was knocked down in the DM group, the density of microvessels increased dramatically (Fig. 6e and 6f).

## DISCUSSION

It is generally accepted that impaired angiogenesis plays a vital role in the process of DM wound healing. Endothelial dysfunction induced by hyperglycemia results in abnormal angiogenesis and is the leading cause of diabetic vascular complications [33, 34]. Moreover, the presence of excessive and persistent inflammation concurrently emerges as a contributing factor for delayed or nonhealing in patients with DM wounds [35]. In the inflammatory phase of DM wound healing, immunosuppression leads to M2 macrophage transformation inhibition and vascular endothelial cell growth factor (VEGF) reduction, which finally cause angiogenesis impaired [36, 37]. Therefore, suppressing inflammatory processes and enhancing vascular formation are efficacious interventions for the management of DM wounds[38].

Our research verified that S100A12 plays an important role in inhibiting DM wound healing. As a member of the proinflammatory protein S100 family, S100A12 is highly expressed specifically in neutrophils and monocytes [32, 39, 40]. Our previous studies revealed that keratinocyte-secreted S100A12 has proinflammatory effects on cells under reduced hydration conditions, activating dermal fibroblasts and resulting in hypertrophic scar formation [22]. As the predominant pathological hallmark of DM wounds, vascular endothelial injury caused by persistent local hyperinflammation, and the weakening of vascular proliferation ultimately delay the healing of DM wound. S100A12 upregulate expression and affinity of the integrin receptor CD11b/CD18 (Mac-1) on neutrophils and facilitate the adhesion of monocytes to the endothelium *in vitro* [41, 42]. Observation of the interaction between S100A12 with the endothelium in inflammatory arthritis supporting the pathogenic relevance of these findings [18, 43]. Through our results, abnormal increase of S100A12 in hyperglycemic environment severely hindered the normal physiological behavior of endothelial cells, knockdown of S100A12 strengthened the migration and adhesion of endothelial cells to each other and promoted the formation of neovascularization.

In our observation, S100A12 is excessively secreted in hyperglycemic environments, where it causes endothelial injury by binding to RAGE and TLR4, which are expressed on the endothelial cell surface, further preventing angiogenesis. Initially, RAGE was identified by its ability to bind advanced glycation end products (AGEs), Long-term hyperglycemia in diabetic patients triggers the process of protein glycosylation, which leads to an increase in AGEs and RAGE. S100A12, also known as EN-RAGE, was found to be the first ligand for RAGE among S100 protein family [44]. Hofmann MA demonstrated that the interaction of S100A12 with RAGE on endothelial activates the NF-κB pathway and upregulates the expression of multiple gene products that contribute to the inflammatory response [44]. Inhibiting S100A12-mediated RAGE activation can reduce vascular inflammation in mice and improve atherosclerosis in mouse arteries [45]. TLR4 was confirmed to upregulate the expression of inflammatory cytokines such as IL-1, IL-6 and IL-8 by inducing the activation of NF-κB in 1997 [46]. Studies have highlighted that TLR4 receptors activate inflammatory responses through NF-kB and MAP kinase pathways, which plays an important role in regulating microvascular endothelial cell permeability [47, 48]. In our study, endothelial cells exhibited functional repair after inhibition of RAGE/TLR4 receptors in a high-glucose environment, illustrating the importance of RAGE and TLR4 in in regulating inflammation and vascular homeostasis.

Transcription factors are among the most essential components of complex regulatory networks within cells, and their ability to control gene expression in response to various signals is critical for maintaining cellular homeostasis [49]. However, alterations in the cellular microenvironment can indeed have a significant impact on the expression and activity of transcription factors [50]. To further explore the source of the increase in S100A12 in hyperglycemic environments, we searched the Jaspar database and found that the transcription factor KLF5 had binding sites in the S100A12 promoter. KLF5 mainly regulates the phenotypic switch, proliferation and vascular remodeling of vascular smooth muscle cells (VSMCs) and is further involved in various vascular diseases, underscoring its important role in maintaining vascular homeostasis [24, 51, 52]. We therefore hypothesized that KLF5 may be the key transcription factor involved in inflammation acceleration, endothelial function changes and wound healing in hyperglycemic conditions. KLF5 was confirmed to be strongly correlated with the direct promotion of S100A12 expression and subsequent endothelial dysfunction under high-glucose conditions in our follow-up experiment.

Mice were used to construct diabetic wounds in our initial studies. In subsequent studies, we found that the S100A8/A9 sequence rather than the S100A12 sequence was present in the mouse gene. As a recognized proinflammatory gene [53], the S100A8/A9 sequence is similar to and partially overlaps the S100A12 sequence [39]. In addition, they are related upstream and downstream. The application of siRNA inhibited the expression of S100A8/A9 in wounds, thus inhibiting inflammation and promoting wound healing [54–56]. To directly demonstrate the role of S100A12 in diabetic wound healing *in vivo*, we constructed diabetic wounds in rabbits that contained the S100A12 sequence. S100A12 was verified to play an important role in the healing process of rabbit ears in our previous studies [22]. Compared with mouse skin, the healing of wounds in rabbit ears mostly relies on basal granulation tissue formation instead of epithelial contraction, and the contralateral ear can be used as a control [57, 58].

## CONCLUSION

In summary, our study identified S100A12 as a vascular injury related factor secreted from epidermal cells under high-glucose conditions that impairs endothelial functions and further leads to delays in healing of wounds in patients with diabetes. We anticipate that inhibiting the expression of S100A12 and its transcription factor KLF5 is a potential therapeutic strategy for improving wound healing in patients with DM.

## Supplementary Information

Below is the link to the electronic supplementary material.

Supplementary file1 (DOCX 18 KB)

## Data Availability

All data supporting the findings of this study are available within the paper and its Supplementary Information.

## Declarations

### Inclusion and Diversity

We support inclusive, diverse, and equitable conduct of research.

## Abbreviations

*DM wound*: Diabetes Mellitus wound
*RAGE*: Receptor of Advanced Glycation Endproducts
*TLR4*: Toll-like Receptor 4
*KLF5*: Krüpple-like Factor 5
*ChIP*: Chromatin immunoprecipitation
*ELISA*: Enzyme-linked Immunosorbent Assay
*pGL3-BV*: PGL3 Basic vector
*FBG*: Fasting Blood Glucose
*SKC*: Stratified Keratinocyte Culture
*HMEC-1*: Human Microvascular Endothelial Cell line-1
*VEGF*: Vascular Endothelial Growth Factor
*AGEs*: Advanced Glycation Endproducts
*eNOS*: Endothelial Nitric Oxide Synthase
*HG*: High Glucose
*Vec*: Vector
*siA12*: SiS100A12
