# Supplementary material for: Targeting S100A12 to Improve Angiogenesis and Accelerate Diabetic Wound Healing
Source: Inflammation. 2024 Jul 2;48(2):633–48. doi: 10.1007/s10753-024-02073-8 (PMC12053334; doi:10.1007/s10753-024-02073-8)
Supplement: Supplementary file 1 — Supplementary file1 (DOCX 18 KB) [file 10753_2024_2073_MOESM1_ESM.docx]

**TABLE 1 S100A12 promoter primer sequences**

| **Gene** | **Primer Sequences** |
| --- | --- |
| S100A12 | 1F: 5'CCA TGT CCG CAC CAG CAC CATC 3',  1R: 5'GGC AAT GCA CGT ATT GGC GTC 3' |
|  | 2F: 5'CTT CAC AGG TGT GGG ACA AAG 3'  2R: 5'TCA TGT GGG AGC GGA GTA GAG 3' |
|  | 3F: 5'AAT CTA TTT TGA AAA CTG ATC 3'  3R: 5'CTC TTC AGT TTT GGC AAA TGG 3' |
|  | 4F: 5'GGT GGG TGG AGT GGG GGT GAT 3'  4R: 5'AGA AAA AAG TTC ACA GAC ACTA 3' |
|  | 5F: 5'CAT ATT ATT TGG GAG AGA ATAG 3'  5R: 5'ATC CTC TCG CCC CAG CCTCC 3' |

**TABLE 2 S100A12 shRNAs Sequences**

| **Direction** | **Sequences** |
| --- | --- |
| Forward | 5’-CCGGGCTTACAAAGGAGCTTGCAAACTC  GAGTTTGCAAGCTCCTTTGTAAGCTTTTTG-3’ |
| Reverse | 5’-AATTCAAAAAGCTTACAAAGGAGCTTGC  AAACTCGAGTTTGCAAGCTCCTTTGTAAGC-3’ |

**TABLE 3 Weight and fasting blood glucose of rabbits during wound healing**

| **Groups** | **Day 0** | | **Day 7** | | **Day 14** | |
| --- | --- | --- | --- | --- | --- | --- |
|  | **R.Wt**  **(kg)** | **FBG**  **(mmol/L)** | **R.Wt**  **(kg)** | **FBG**  **(mmol/L)** | **R.Wt**  **(kg)** | **FBG**  **(mmol/L)** |
| A | 1.98±0.12 | 5.71±0.38 | 2.21±0.12 | 5.98±0.62 | 2.31±0.09 | 5.57±0.41 |
| B | 1.99±0.11 | 5.56±0.31 | 2.27±0.09 | 24.75±1.50* | 2.29±0.16 | 24.42±1.83* |
| C | 2.01±0.10 | 5.83±0.51 | 2.24±0.12 | 25.06±1.77* | 2.30±0.17 | 25.75±1.31* |
| D | 2.02±0.08 | 5.67±0.36 | 2.21±0.11 | 25.24±0.96* | 2.30±0.10 | 24.39±1.89* |

Abbreviation: R.Wt: Rabbit Weight; FBG: Fasting Blood Glucose; DM: Diabetic Mellitus; Vec: Vector; siA12: siS100A12.

Group A: Normal group; Group B: DM group; Group C: DM+Vec group; Group D: DM+siA12 group. Values are mean±SD, *P<0.05 vs Normal group, n=6.
